# Influence of nonsteroidal anti-inflammatory drugs on aspirin’s antiplatelet effects and suggestion of the most suitable time for administration of both agents without resulting in interaction

**DOI:** 10.1186/s40780-017-0078-7

**Published:** 2017-03-09

**Authors:** Kenta Shibata, Yuuki Akagi, Naofumi Nozawa, Hitoshi Shimomura, Takao Aoyama

**Affiliations:** 1 Faculty of Pharmaceutical Sciences, Tokyo University of Science, 2641 Yamazaki, Noda, Chiba 278-8510 Japan; 2 Department of Pharmacy, Koshigaya Municipal Hospital, 10-47-1 Higashi-Koshigaya, Koshigaya, Saitama 343-0023 Japan; 3 Department of Pharmacy, National Hospital Organization, Yokohama Medical Center, 3-60-2 Harajuku, Totsuka, Yokohama, Kanagawa 245-8575 Japan; 4 Department of Pharmacy, Chemotherapy Research Institute, Kaken Hospital, 6-1-14 Konodai, Ichikawa, Chiba 272-0827 Japan

**Keywords:** Aspirin, Antiplatelet, Nonsteroidal anti-inflammatory drugs, Ibuprofen, Loxoprofen, Drug interaction, Human, Cyclooxygenase-1, Coating, Platelet aggregation threshold index

## Abstract

**Background:**

Low-dose aspirin irreversibly inhibits platelet cyclooxygenase-1 (COX-1) and suppresses platelet aggregation. It is effective for secondary prevention of cardiovascular events. Because nonsteroidal anti-inflammatory drugs (NSAIDs) reversibly bind with COX-1, the antiplatelet effects of aspirin may be suppressed when NSAIDs are co-administered. This interaction could be avoided by avoiding simultaneous administration; however, the minimum interval that should separate the administration of aspirin and loxoprofen is not well known. In this study, we investigated how to avoid the influence of NSAIDs on the antiplatelet effects of aspirin. An in vitro experiment was performed to investigate the influence of ibuprofen and loxoprofen at various concentrations on aspirin’s antiplatelet action.

**Methods:**

Platelet aggregation and thromboxane B_2_ (TXB_2_) levels were measured after addition of aspirin only and NSAIDs plus aspirin to platelet-rich plasma. NSAIDs were used at their maximum plasma concentrations, the assumed concentration after 6 h (for loxoprofen only), and the assumed concentration after 12 h of taking one clinical dose. Platelet aggregation threshold index (PATI), defined as the putative stimulus concentration giving 50% aggregation, was calculated as an index of aggregation activity.

**Results:**

PATI decreased in ibuprofen plus aspirin group compared to that in the aspirin only group, regardless of ibuprofen concentration. Furthermore, PATI significantly decreased when aspirin was added after loxoprofen-*trans*-OH addition at the maximum concentration (4.1 ± 0.1 μg/mL), compared to that in aspirin only group (5.9 ± 0.1 μg/mL). PATI showed no significant difference after addition of loxoprofen at the assumed concentration after 6 h (aspirin only group, 5.0 ± 0.5 μg/mL; loxoprofen-*trans*-OH plus aspirin group, 4.9 ± 0.4 μg/mL).In addition, TXB_2_ concentration tended to decrease with increasing PATI.

**Conclusions:**

It is desirable to avoid ibuprofen co-administration with the usual once-daily low-dose aspirin therapy; however, a 6-h interval between loxoprofen and aspirin could avoid this potential interaction when loxoprofen is taken before aspirin.

## Background

Since low-dose aspirin was found to suppress platelet aggregation [1], several clinical trials have demonstrated its antiplatelet efficacy [2]. Low-dose aspirin is widely used for angina pectoris, myocardial infarction, ischemic cerebro-vascular disease, Kawasaki disease, and for prevention of thromboembolism after cardiac surgery. It inhibits cyclooxygenase-1 (COX-1) in the platelets, suppresses arachidonic acid metabolism, and prevents the synthesis of thromboxane A_2_ (TXA_2_), a compound that induces platelet aggregation [3]. Aspirin is considered to suppress platelet aggregation by acetylating platelet COX-1 in portal immediately after taking the medication [4]. Aspirin acts on the internal COX-1 and irreversibly acetylates Ser529 [3]. Access to COX-1 active site, responsible for TXA_2_ synthesis, is then impeded for the lifetime of the platelet. When COX-1 in the platelets is acetylated by aspirin, the antiplatelet effects of aspirin are suggested to depend on platelet turnover and to be maintained until new platelets, unacetylated by aspirin, are produced [5].

Nonsteroidal anti-inflammatory drugs (NSAIDs), including ibuprofen and loxoprofen, are widely used as analgesics, antipyretics, and anti-inflammatory agents [6]. NSAIDs also inhibit the access to COX-1 active site for TXA_2_ synthesis; however, the mechanism of inhibition involves formation of a salt bridge with Arg120 of COX-1, unlike aspirin [7]. Suppression of the platelet function by NSAIDs is limited to a fixed period after their administration, because COX-1 inhibition is reversible [8].

Some patients with cardiovascular disorders using low-dose aspirin simultaneously take NSAIDs for relief of pain resulting from conditions, such as rheumatoid arthritis [9]. The antiplatelet effects of aspirin may be decreased due to co-administration of ibuprofen [3, 10, 11], and a warning is included in the package inserts of both aspirin and ibuprofen. When ibuprofen binds with COX-1, it hinders acetylation of the serine residue by aspirin [3]. Therefore, aspirin needs to bind with COX-1 before ibuprofen administration to avoid this interaction. It has been reported that the reduction of the antiplatelet effects of aspirin was avoided by taking aspirin 2 h before ibuprofen administration [3]. Although this dosage regimen is only useful in case of single daily dose of aspirin and ibuprofen, it is considered that the antiplatelet effects of daily low-dose aspirin (once a day) are competitively inhibited by the prolonged use of multiple daily doses of ibuprofen (three times a day), even when aspirin was taken before ibuprofen administration [3, 10, 11]. COX-1 inhibition seems to persist when aspirin is taken on the next day, in spite of the 12-h period since the last dose of ibuprofen.

Loxoprofen sodium is more frequently prescribed than ibuprofen in Japan [9, 12]. The antiplatelet effects of aspirin were found to be suppressed by loxoprofen; however, we previously reported that this interaction might be avoided by taking aspirin 2 h before loxoprofen in continuous dosing [13]. In other words, this interaction might be avoided by taking aspirin 12 h after loxoprofen administration.

Some patients take NSAIDs irregularly in case of pain and self-adjustment. Thus, it seems to be difficult to secure 12 h to avoid this interaction. The minimum time interval that should separate the administration of aspirin and loxoprofen to avoid their interaction is not well known. In addition, there are two forms of aspirin tablets, uncoated and enteric-coated tablets. Enteric-coated aspirin tablets result in a slower inhibitory effect on platelet COX-1 than the uncoated aspirin tablets [14]. Therefore, NSAIDs might have different effects depending on the dosage form of aspirin.

We previously reported that the antiplatelet effect of aspirin was inhibited by ibuprofen but not the other five NSAIDs investigated in an in vitro experimental study [15]. In this report, the final concentrations of the NSAIDs were their maximum plasma concentrations (C_max_) obtained after one clinical dose. We considered that the antiplatelet effect of the NSAIDs might apparently conceal their interaction with aspirin. The extent to which the antiplatelet effect of aspirin was affected was thought to be depended on the COX-1 inhibitory activity of each NSAID.

In this study, we investigated that the time interval required to avoid the influence of NSAIDs on the antiplatelet effects of aspirin. An in vitro experiment was performed, in which aspirin and NSAIDs were added to human blank blood, to investigate the influence of NSAIDs at various concentrations on the antiplatelet action of aspirin. The fluctuation of the serum drug concentration with time was considered. We washed the platelet to avoid the antiplatelet effect of the NSAIDs, which apparently could affect their interaction with aspirin.Two NSAIDs, ibuprofen and loxoprofen, were used. Some information is known about the interaction of ibuprofen with aspirin; however, only limited information is known in case of loxoprofen. Moreover, we investigated the best timing for taking aspirin, in case of both uncoated and enteric-coated tablets, and NSAIDs to avoid the reduction of the antiplatelet effects of low-dose aspirin.

## Methods

### Chemicals

Aspirin (Lot. No. TSH5399), ibuprofen (Lot. No. TSG6469 and KWF6951) were obtained from Wako Pure Chemical Industries (Osaka, Japan). Loxoprofen-*trans*-OH (a mixture of four isoforms of *trans*-OH loxoprofen metabolites, Lot. No. L-0179-046-T) was kindly provided by Daiichi Sankyo Company (Tokyo, Japan). HEPES (Lot. No. EX146, EQ115, CW082, and DB223) was obtained from Dojindo Laboratories (Kumamoto, Japan). Dimethyl sulfoxide (DMSO, Lot. No. 911 W1022 and 107 N1039) was obtained from Kanto Chemical Industries (Tokyo, Japan). Human plasma albumin (Lot. No. M9G6056 and M2M2762) was obtained from Nacalai tesque (Kyoto, Japan). Collagen (Lot. No. C003, 0538, 0540, and 0543), as a platelet aggregation stimulus, was obtained from Arkray (Kyoto, Japan). All other chemicals were of analytical grade.

### Blood collection

Blood samples were collected from healthy adult volunteers, including both men and women (*n* = 6, however *n* = 5 for groups added the concentration after 12 h of taking one clinical dose). Inclusion criteria were non-smokers, not taking aspirin within 2 weeks or other medications (containing NSAIDs) within 1 week before blood collection, and avoiding alcohol, fatty meals, and large amounts of spinach, garlic, Chinese chive, and onion after the evening meal on the day before blood collection because they may affect platelet aggregation activity [16, 17]. About 20 mL of blood was collected using a 21G needle into a plastic syringe containing 2 mL of acid-citrate-dextrose (ACD)-A solution. The blood was divided into plastic tubes containing 5 mL each and centrifuged at 200 *g* for 10 min at 25 °C. Platelet rich plasma (PRP) was obtained from the supernatant of the blood. Study protocols were approved by the ethics committee for human material analysis study of the Tokyo University of Science (approval number 10001, 13002).

### Addition of drugs to platelets

Aspirin, ibuprofen and loxoprofen-*trans*-OH were suspended in a little volume of DMSO, diluted with saline containing 0.1% human plasma albumin and 1% DMSO (0.1% Alb) [18]. The final concentration of aspirin was 17 μM, which was enough to induce antiplatelet effects in the in vitro experimental protocol that adding drugs to PRP. When the final concentration of aspirin was lower than 17 μM, the PATI values were not steady. In contrast, when the final concentration of aspirin was much higher than 17 μM, platelet aggregation was hardly induced by the highest concentration of collagen (10 μg/mL). Therefore, it was impossible to measure the PATI values. The final concentrations of NSAIDs (ibuprofen and loxoprofen-*trans*-OH) were their C_max_, the concentration after 6 h (for loxoprofen only), and the concentration after 12 h of taking one clinical dose (ibuprofen, 200 mg; loxoprofen, 60 mg, Table 1) [19, 20]. The concentration of NSAIDs after 12 h is assumed to be equal to that before taking aspirin (at 7:00 in the next morning) in a dosage regimen involving NSAID administration 3 times a day (at 9:00, 13:00, and 19:00). A control, aspirin only, and NSAID plus aspirin groups were studied for each NSAID. In NSAID plus aspirin group, aspirin was added to PRP 6 min after NSAID addition. Each fluid volume was 1 mL (PRP), 111 μL (NSAID solution), and 123 μL (aspirin solution). In aspirin only group, 0.1%Alb was added instead of the NSAID while in the control group, 0.1%Alb was added instead of aspirin and NSAID. Because platelet COX-1 was inhibited by aspirin or NSAIDs within 5 min [21] in an in vitro experimental study and platelet aggregation rate may be fluctuant if the elapsed time after aspirin addition was not constant; therefore, preparation of washed platelets started 6 min after aspirin addition.

**Table 1:** Concentration of each drug in the additional experiment

Drug	Concentration (μM)
Aspirin	17
Ibuprofen (maximum plasma concentration after taking ibuprofen 200 mg)	80.5 [19]
Ibuprofen (assumed concentration after 12 h of taking ibuprofen 200 mg)	2 [19]^a^
Loxoprofen-*trans*-OH (maximum plasma concentration after taking loxoprofen 60 mg)	3.4 [20]
Loxoprofen-*trans*-OH (assumed concentration after 6 h of taking loxoprofen 60 mg)	0.16 [20]
Loxoprofen-*trans*-OH (assumed concentration after 12 h of taking loxoprofen 60 mg)	0.0046 [20]

^a^Concentration was simulated using a 1-compartment model

### Measurement of platelet aggregation

Plt-HEPES solution containing 1 mM MgCl_2_, 20 mM HEPES, 0.13 M NaCl, 2 mM KCl, and 0.4 mM NaH_2_PO_4_ was prepared. Then, the PRP-drug solution mixture with added buffer (Plt-HEPES solution: ACD-A solution = 9: 1) was centrifuged at 25 °C, 520 *g* for 10 min, and the obtained platelet pellets were re-suspended in Plt-HEPES solution and CaCl_2_ solution was added (final concentration of CaCl_2_ was 2 mM).

Platelet aggregation was measured using a light transmission aggregometer, PRP313M (IMI Co., Ltd., Saitama, Japan), according to the Born and Cross method [22]. One-hundred microliters of platelet suspension were pre-incubated in a cuvette at 37 °C for 3 min in the PRP313M. Platelet aggregation was measured using collagen as a stimulus for 5 min. The final concentrations of collagen in the platelet suspension were 0.5, 2, 5, and 10 μg/mL. Assuming the light transmission through the buffer to be 100%, that of the platelet suspension represented the platelet aggregation rate. Platelet aggregation was measured within 1 to 2 h after blood collection because the platelet aggregability was stable during this period [23].

### Data analysis

#### 1) Platelet aggregation rate

**Fig. 1:**
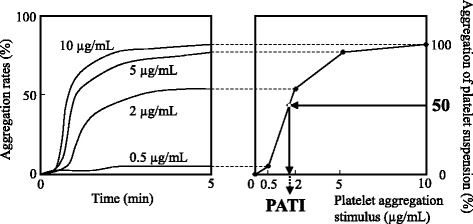
Calculation method of platelet aggregation threshold index (PATI [24]). Light transmission curves were obtained from four experiments (collagen concentration, A: 0.5, B: 2, C: 5, and D: 10 μg/mL)

The maximum aggregation rates induced by collagen at the four concentrations used were connected as a grading curve. The maximum aggregation rate induced by the highest concentration of collagen (10 μg/mL) was defined as 100% of platelet suspension, and the putative agonist-concentration giving 50% aggregation was calculated and defined as platelet aggregation threshold index (PATI, Fig. 1) [24]. Therefore, the increase in PATI means that the platelet aggregation activity was inhibited because a higher concentration of the stimulus was needed to induce platelet aggregation. The PATI values represent the mean ± SEM.

Besides, there are inter-individual and intra-individual differences in the platelet aggregation activity [25], thus we also measured platelet aggregation in a drug-free group (0.1% Alb) as a control in each additional condition.

#### 2) Measurement of thromboxane B_2_ (TXB_2_) level in platelet suspension

Platelet COX-1 activity was determined by measuring serum levels of TXB_2_, the major stable metabolite of TXA_2_. TXB_2_ level in platelet suspension was determined using enzyme-linked immunosorbent assay (Thromboxane B_2_ EIA Kit, Cayman Co., Ann Arbor, Michigan, USA). To measure TXB_2_ derived from platelet COX-1, TXB_2_ concentration in the supernatant of the collagen-stimulated platelet suspension was determined [26]. Platelet suspension was stimulated with 2 or 5 μg/mL collagen for 5 min, followed by centrifugation at 4 °C, 2000 *g* for 15 min to remove platelets. Supernatants were stored at −30 °C until analysis.

#### 3) Statistical analysis

Tukey’s test was used to compare PATI and TXB_2_ concentrations in platelet suspension in each condition. *P* value < 0.05 is considered statistically significant.

## Results

### Added NSAIDs: ibuprofen

**Fig. 2:**
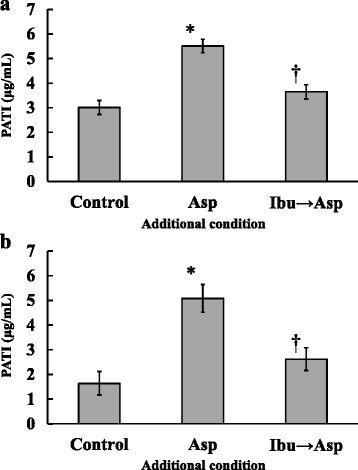
Effect of aspirin (Asp) and aspirin after ibuprofen (Ibu → Asp) addition on platelet aggregation threshold index (PATI). Ibuprofen concentration of (**a**): maximum concentration after taking ibuprofen at a dose of 200 mg, (**b**): the concentration 12 h after taking ibuprofen at a dose of 200 mg. (*n* = 6 for a, *n* = 5 for b; *P < 0.05 compared with the control; †*P* < 0.05 compared with the aspirin only group; These values represent the mean ± SEM.)

**Fig. 3:**
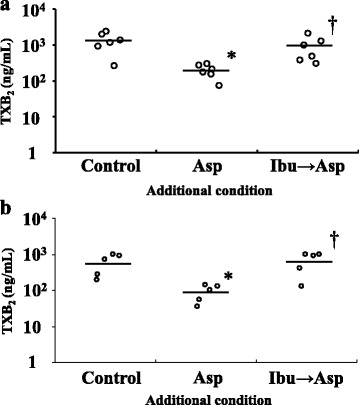
TXB_2_ concentration of aspirin (Asp) and aspirin after ibuprofen (Ibu → Asp) addition. Ibuprofen concentration of (**a**): maximum concentration after taking ibuprofen at a dose of 200 mg, (**b**): the concentration 12 h after taking ibuprofen at a dose of 200 mg. (*n* = 6 for a, *n* = 5 for b; *P < 0.05 compared with the control; †*P* < 0.05 compared with the aspirin only group; The line indicates the mean value.)

The PATI values of the control and ibuprofen (C_max_) plus aspirin groups were 3.0 ± 0.3 and 3.7 ± 0.3 μg/mL, respectively. These values were lower than that of the aspirin only group (5.5 ± 0.3 μg/mL, Fig. 2a). TXB_2_ concentrations in platelet suspension of the control, aspirin only, and ibuprofen (C_max_) plus aspirin groups were 1400 ± 300, 200 ± 30, and 930 ± 290 ng/mL, respectively, which decreased with increasing PATI (Fig. 3a). When ibuprofen was used at the assumed concentration after 12 h of taking one clinical dose, PATI of the control, aspirin only and ibuprofen plus aspirin groups were 1.6 ± 0.5, 5.1 ± 0.6, and 2.6 ± 0.5 μg/mL, respectively (Fig. 2b). TXB_2_ concentrations in platelet suspension of the control, aspirin only and ibuprofen plus aspirin groups were 580 ± 150, 85 ± 19, and 650 ± 170 ng/mL, respectively, which decreased with increasing PATI (Fig. 3b). PATI in the ibuprofen plus aspirin group was lower than that in the aspirin only group, regardless of the concentration of ibuprofen. Additionally, the PATI values and TXB_2_ concentrations of the platelet suspension of the group treated with ibuprofen only were nearly equal to those of each control group, regardless of their concentrations (data not shown).

### Added NSAIDs: loxoprofen-*trans*-OH

**Fig. 4:**
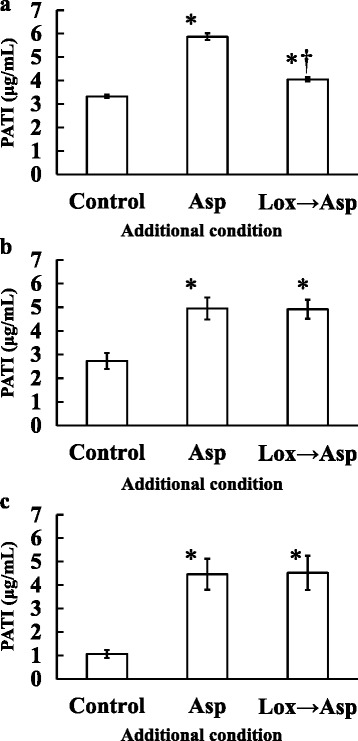
Effect of aspirin (Asp) and aspirin after loxoprofen-*trans*-OH (Lox → Asp) addition on platelet aggregation threshold index (PATI). Loxoprofen-*trans*-OH concentration of (**a**): maximum concentration after taking loxoprofen at a dose of 60 mg, (**b**): the concentration 6 h after taking loxoprofen at a dose of 60 mg, (**c**): 12 h after taking loxoprofen at a dose of 60 mg (*n* = 6 for a and b, *n* = 5 for c; *P < 0.05 compared with the control; †*P* < 0.05 compared with the aspirin only group; These values represent the mean ± SEM.)

**Fig. 5:**
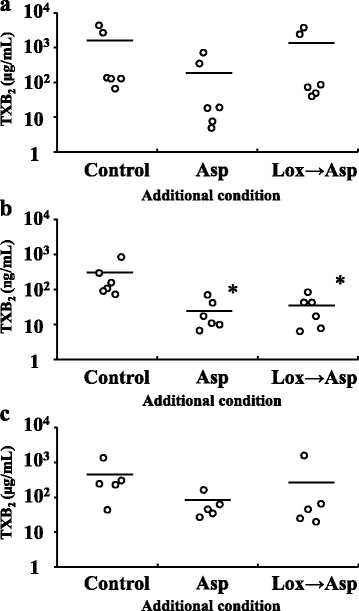
TXB_2_ concentration of aspirin (Asp) and aspirin after loxoprofen-*trans*-OH (Lox → Asp) addition. Loxoprofen-*trans*-OH concentration of (**a**): maximum concentration after taking loxoprofen at a dose of 60 mg, (**b**): the concentration 6 h after taking loxoprofen at a dose of 60 mg, (**c**): the concentration 12 h after taking loxoprofen at a dose of 60 mg (*n* = 6 for a and b, *n* = 5 for c; *P < 0.05 compared with the control; The line indicates the mean value.)

The PATI values of the aspirin only and loxoprofen-*trans*-OH (C_max_) plus aspirin groups were 5.9 ± 0.1 and 4.1 ± 0.1 μg/mL, respectively. These values were higher than that of the control (3.3 ± 0.1 μg/mL, Fig. 4a). PATI of the loxoprofen-*trans*-OH (C_max_) plus aspirin group was significantly decreased compared to that of the aspirin only group. TXB_2_ concentrations in platelet suspension of the control, aspirin only, and loxoprofen-*trans*-OH (C_max_) plus aspirin groups were 1200 ± 700, 190 ± 120, 1100 ± 700 ng/mL, respectively (Fig. 5a). Each data showed that TXB_2_ concentrations decreased with increasing PATI; however, two of the six subjects showed scores that were several times higher than the TXB_2_ concentrations of the others. When loxoprofen was used at the assumed concentration after 6 h of taking a clinical dose of loxoprofen sodium, PATI of the aspirin only and loxoprofen-*trans*-OH plus aspirin groups were 5.0 ± 0.5 and 4.9 ± 0.4 μg/mL, respectively. These values were higher than that of the control (2.7 ± 0.3 μg/mL, Fig. 4b). In addition, TXB_2_ concentrations in the aspirin only and loxoprofen-*trans*-OH plus aspirin groups were 26 ± 10 and 33 ± 12 ng/mL, respectively, which was lower than that of the control (260 ± 120 ng/mL, Fig. 5b). PATI and TXB_2_ concentrations in platelet suspension showed no significant differences between the aspirin only and loxoprofen-*trans*-OH plus aspirin groups. Similar results were obtained when loxoprofen was used at the assumed concentration after 12 h of taking a clinical dose of loxoprofen sodium (Figs. 4c and 5c). Additionally, the PATI values and TXB_2_ concentrations of the platelet suspension of loxoprofen-*trans*-OH only groups were nearly equal to those of each control group, regardless of their concentrations (data not shown).

## Discussion

The antiplatelet effects of aspirin were suppressed when aspirin was added after ibuprofen, regardless of the concentration of ibuprofen. TXB_2_ level in platelet suspension in the ibuprofen plus aspirin group was higher than that in the aspirin only group. Platelet COX-1 inhibition by aspirin was considered to be decreased (Fig. 3a and b). Besides, PK/PD analysis showed that taking aspirin 2 h before ibuprofen can avoid this interaction, whereas taking ibuprofen 12 h before aspirin cannot avoid this interaction [27]. The binding constant of ibuprofen to COX-1 (2.48 μM^−1^.hr^−1^) is larger than that of aspirin (0.027 μM^−1^.hr^−1^) [28], and the concentration of ibuprofen 12 h after taking the medication (about 2 μM) is nearly equal to the half maximal inhibitory concentration (IC_50_) of ibuprofen to COX-1 (3.0 – 4.8 μM) [29–31]. This concentration seems to be enough to inhibit the antiplatelet effects of aspirin. Regarding the usual once-daily low-dose aspirin regimen, it is difficult to avoid the interaction between aspirin and ibuprofen because the antiplatelet effects of aspirin are suppressed by ibuprofen even if aspirin is taken 12 h after ibuprofen administration. It is possible to avoid this interaction when the dosing interval from ibuprofen to aspirin is 22 h (in other words, from aspirin to ibuprofen is 2 h) in this once-daily regimen [3]; however, it is a considerably limited condition. Thus, it is desirable to avoid taking ibuprofen by patients on low-dose aspirin therapy.

Many studies focused on the interaction between aspirin and ibuprofen because ibuprofen is generally prescribed worldwide [6, 32]. However, since loxoprofen sodium is frequently prescribed in Japan [9, 12], and its price is low, loxoprofen sodium is likely to be involved in the case of taking low-dose aspirin and NSAIDs concomitantly in Japan.

Loxoprofen sodium is metabolized into four isomers of loxoprofen-*trans*-OH (loxoprofen-RSS, SRS, RSR, and SRR) in the human body [33]. Only loxoprofen-SRS has antipyretic, analgesic, and anti-inflammatory action, and inhibit platelet COX-1 [34]. Though the percentage of the SRS-isomer in loxoprofen-*trans*-OH used in our study was unclear, it was considered one quarter. The antiplatelet effects of aspirin were suppressed in when loxoprofen-*trans*-OH was added at C_max_, whereas it was not suppressed when loxoprofen-*trans*-OH was added at the assumed concentration after 6 h (Fig. 4a and b). TXB_2_ level in platelet suspension of the loxoprofen-*trans*-OH (C_max_) plus aspirin group tended to be higher than that of the aspirin only group. All the subjects exhibited the same tendency although no significant difference was shown (Fig. 5a). However, TXB_2_ was not high at the assumed loxoprofen-*trans*-OH concentration after 6 h of taking loxoprofen dose (Fig. 5b). Therefore, the antiplatelet effects of aspirin were affected by loxoprofen; however, taking aspirin 6 h after loxoprofen administration could avoid this interaction. The theoretical concentrations of loxoprofen-SRS are about 0.85 μM (C_max_) and 0.04 μM (after 6 h of taking loxoprofen). The C_max_ of loxoprofen-SRS after a single oral dose of loxoprofen tablet (60 mg) was about 1.17 μM [33]. The IC_50_ of loxoprofen-SRS to platelet COX-1 was about 0.38 μM [29]. Therefore, the C_max_ of loxoprofen-*trans*-OH was enough to inhibit platelet COX-1 although this inhibition was reversible, whereas loxoprofen-*trans*-OH concentration after 6 h of taking loxoprofen might be too low to inhibit COX-1. Therefore, when loxoprofen-*trans*-OH was added at the C_max_, it competitively impeded COX-1 acetylation by aspirin when NSAIDs reach the active site on platelet COX-1 earlier than aspirin [3]. Considering the theoretical concentrations of loxoprofen-SRS, platelet COX-1 was difficult to be inhibited by loxoprofen-SRS 6 h or more after taking loxoprofen, thus this interaction can be avoided. Loxoprofen is eliminated rapidly [20], so the effect of loxoprofen on the antiplatelet effect of aspirin was thought to decrease within 6 h of taking loxoprofen. The theoretical concentration of loxoprofen-SRS 4 h after dosing (approximately 0.14 μM) was lower than its IC_50_ against platelet COX-1 (approximately 0.38 μM) [20, 29]. However, we considered that the platelet COX-1 might have been partially inhibited because the ratio of its IC_50_ to the concentration was only 2.7, which might be the border line at which the interaction was observed or not. Therefore, a dosing interval of 6 h from when loxoprofen is administered to when aspirin is administered was considered sufficient, but it is unclear whether the dosing interval of 6 h is necessary or not. Further studies are needed to clarify whether a dosing interval from when loxoprofen is administered to when aspirin is administered is possible to be reduced less than 6 h with avoiding this interaction.

In this study, the concentration of each NSAID was determined based on the clinical single dose. Ibuprofen and loxoprofen can be taken three times a day. However, ibuprofen and loxoprofen are eliminated rapidly; therefore, they do not accumulate in the body even when they are taken continuously [35].

**Fig. 6:**
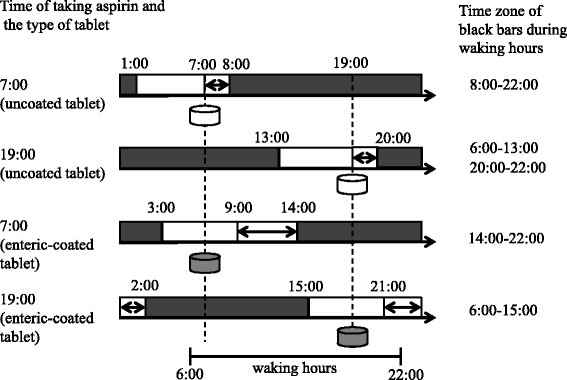
Avoidable time zone in the four model cases (the time of taking aspirin and the coating of aspirin tablet are different) of taking aspirin with loxoprofen (shown as *black bars*). The double-headed arrows indicate the falling time zone of TXB_2_. In these model cases, the waking time is 6:00 to 22:00

We studied NSAID interference with the antiplatelet effects of aspirin via COX-1 inhibitory activity of NSAIDs; however, it is necessary to consider the rate of COX-1 irreversible inhibition by aspirin, because two forms of aspirin tablets, uncoated and enteric-coated, with different solubility are widely used in Japan. Aspirin is metabolized to salicylic acid and its half-life in human plasma is approximately 5–15 min [36]. Platelet COX-1 is rapidly acetylated by aspirin in the portal vein during this metabolism period. This time zone was determined based on the decline in serum TXB_2_ concentration. A sudden decline in serum TXB_2_ concentration occurs 0 to 1 h after taking uncoated aspirin tablets [14, 37], and it is of great importance that NSAIDs bind to COX-1 and impede its acetylation by aspirin within this one hour. In other words, unless loxoprofen-SRS binding time zone to COX-1 overlaps with the COX-1 acetylation time zone by aspirin, this interaction might be avoided. Considering platelet COX-1 inhibition by loxoprofen-SRS, if loxoprofen is not administered within 6 h before or 1 h after (total 7 h) taking aspirin, loxoprofen will not suppress the antiplatelet effects of uncoated aspirin tablets (Fig. 6). On the other hand, a slow decrease in serum TXB_2_ concentration occurs 2 to 7 h after taking enteric-coated aspirin tablets [14]. Thus, if loxoprofen is not administered within 4 h before or 7 h after (total 11 h) taking aspirin, loxoprofen will not interfere with the antiplatelet effects (Fig. 6). Considering a lifestyle of getting up at 6:00, going to bed at 22:00 and taking aspirin at 7:00 or 19:00, the longest time period in which the interaction can be avoided is achieved by administration of aspirin uncoated tablets at 7:00, which results in a period of 14 h (from 8:00 to 22:00) during which the interaction can be avoidable. Thus, uncoated aspirin tablets are useful to prevent this interaction when NSAIDs are taken several times daily. As a supplement, some studies reported that gastric toxicity from aspirin could be eliminated by the use of enteric-coated tablets; however most of them were short-time studies [38, 39]. Others reported that enteric-coating might have little clinical benefits reducing gastrointestinal bleeding or ulceration longitudinally [40, 41]. Therefore, enteric-coated aspirin is considered to be not always useful to avoid gastric toxicity.

In contrast to ibuprofen and loxoprofen, celecoxib is known as a selective COX-2 inhibitor. No reduction of the antiplatelet effects of aspirin was observed with celecoxib [10]. There are several clinical trials that report the interaction between low-dose aspirin and celecoxib when uncoated aspirin tablets are used in some cases; however, there are no reports that is specified the use of enteric-coated aspirin. Celecoxib IC_50_ to platelet COX-1 was 6.7–8.3 μM [30, 31], and the maximum plasma concentration of celecoxib after a single oral dose of 200 mg is about 2.1 μM. Celecoxib is not considered to interfere with the inhibition of platelet COX-1 by aspirin. Celecoxib was not investigated in our study; however, COX-1 inhibition by celecoxib is considered limited. It is suggested that celecoxib does not suppress the antiplatelet effects of aspirin, regardless of the kind of tablet coating.

These findings suggest that NSAIDs can influence the antiplatelet effects of aspirin depending on the time of taking both drugs and the coating of aspirin tablets. Suppression of the antiplatelet effects may lead to inefficient prevention of cardiovascular events. In fact, patients receiving aspirin plus ibuprofen showed an increased risk of cardiovascular mortality compared with those who received aspirin alone [42]. Both ibuprofen and loxoprofen are used as both ethical and over the counter drugs in Japan, thus pharmacists need to counsel the patients about the time of taking NSAIDs, including nonprescription drugs, with low-dose aspirin.

When low-dose aspirin and NSAIDs are co-administered, several reports showed that ibuprofen suppressed the antiplatelet effects of aspirin; however, celecoxib did not [3, 10, 11]. In addition, loxoprofen suppressed the antiplatelet effects when co-administered with aspirin at the same time; however, this interaction is avoidable [13]. Moreover, the extent to which this interaction is avoidable depends on the time of taking aspirin and loxoprofen, and the coating of aspirin tablets. In this model, the longest period (from 8:00 to 22:00) during which the interference with the antiplatelet effects of aspirin is avoidable, is achieved when uncoated aspirin tablets are administered at 7:00 in the morning.

## Conclusions

It is desirable to avoid ibuprofen co-administration with the usual once-daily low-dose aspirin therapy; however, a 6-h interval between loxoprofen and aspirin could avoid this potential interaction when loxoprofen is taken before aspirin.

## Abbreviations

ACD: Acid-citrate-dextrose
COX-1: Cyclooxygenase-1
IC_50_: The half maximal inhibitory concentration
NSAIDs: Nonsteroidal anti-inflammatory drugs
PATI: Platelet aggregation threshold index
PRP: Platelet rich plasma
TXA_2_: Thromboxane A_2_
TXB_2_: Thromboxane B_2_
