# Erratum to: Influence of nonsteroidal anti-inflammatory drugs on aspirin’s antiplatelet effects and suggestion of the most suitable time for administration of both agents without resulting in interaction

**DOI:** 10.1186/s40780-017-0079-6

**Published:** 2017-04-04

**Authors:** Kenta Shibata, Yuuki Akagi, Naofumi Nozawa, Hitoshi Shimomura, Takao Aoyama

**Affiliations:** 1 Faculty of Pharmaceutical Sciences, Tokyo University of Science, 2641 Yamazaki, Noda, Chiba 278-8510 Japan; 2 Department of Pharmacy, Koshigaya Municipal Hospital, 10-47-1 Higashi-Koshigaya, Koshigaya, Saitama 343-0023 Japan; 3 Department of Pharmacy, National Hospital Organization, Yokohama Medical Center, 3-60-2 Harajuku, Totsuka, Yokohama, Kanagawa 245-8575 Japan; 4 Department of Pharmacy, Chemotherapy Research, Institute, Kaken Hospital, 6-1-14 Konodai, Ichikawa, Chiba 272-0827 Japan

## Erratum

Following publication of this article [1], it has come to our attention that Fig. 5a and c should have been displayed as "ng/mL" rather than “μg/mL”.
